# Frequency and distribution of dietary energy, vegetable, fruit and discretionary food intakes in 18-month-old Australian children

**DOI:** 10.1017/S0007114522003324

**Published:** 2023-08-14

**Authors:** Jie Min Chui, Kathleen E. Lacy, Miaobing Jazzmin Zheng, Rebecca M. Leech, Sarah A. McNaughton, Alison C. Spence

**Affiliations:** 1 Deakin University, School of Exercise and Nutrition Sciences, Geelong, Australia; 2 Deakin University, Institute for Physical Activity and Nutrition (IPAN), School of Exercise and Nutrition Sciences, Geelong, Australia

**Keywords:** Toddler, Pre-schooler, Young children, Intake distribution

## Abstract

Dietary behaviours in early childhood are understudied despite links with later health. Assessing the distribution of key food groups across the day could identify opportunities for improvements. This study aimed to describe the 24-hourly distribution of dietary intakes and frequency of eating occasions for weekdays and weekend days among children aged 18 months and assess associations of eating frequency with vegetable, fruit and discretionary intakes and zBMI. Using two parent-reported 24-h recalls of child dietary intakes from the Melbourne Infant Feeding Activity and Nutrition Trial (InFANT) Program, mean frequency of daily eating occasions and hourly intake distributions were calculated for vegetables, fruits, discretionary foods, and total foods and energy-containing beverages on weekdays (*n* 428) and weekend days (*n* 376). Multivariable regression analyses assessed associations between frequency of eating occasions, total intake of food groups and zBMI. Overall, children had 7·8 ± 1·8 (mean ± sd) eating occasions/d on weekdays, where 1·5 ± 0·8 contained vegetables, 2·2 ± 1·1 contained fruit and 2·5 ± 1·5 contained discretionary foods. Weekend day intakes were similar. Energy intakes were highest at dinner time. Intakes of total foods, fruits and discretionary foods were spread across the day (06.00–22.00). Vegetable consumption was mainly about 18.00 with minimal intake at other times. Eating frequency was associated with amount of food consumed but not consistently with zBMI. These 18-month-old children ate frequently throughout the day, with little distinction between weekdays and weekend days. Most eating occasions lacked vegetables, and frequency of discretionary foods was higher than of vegetables. Promoting vegetable consumption at occasions other than dinner could improve vegetable intake.

Toddlerhood reflects an important dietary transition between milk-based nutrition and family foods. Despite the significance of this transition, national surveys of dietary intakes of children below the age of 2 years are scarce compared with other age groups^(1)^. Available data suggest that few young children meet vegetable recommendations, and the proportion meeting fruit recommendations diminishes across early years^(2,3)^. Additionally, discretionary food intakes (energy-dense, nutrient-poor foods not essential in a healthy dietary pattern) are high from as young as 12–18 months of age^(4)^. This is of concern because intakes of discretionary foods increase and track across child ages^(2)^ and are associated with obesity and risk of developing chronic conditions, such as metabolic syndrome and poor mental health, as children grow^(5)^. Vegetables, fruit and discretionary intakes are the focus of this study as they are the main food groups impacting dietary energy density^(6,7)^ and are considered key influences on the prevalence of chronic diseases^(8,9)^, and consumption in other age groups is most disparate from recommendations^(10)^. Given the importance of the first 1000 d for both short-term and longer-term health, targeting early childhood diets could influence preference for nutrient-rich foods, leading to improved intakes and health across subsequent life stages^(11)^.

Given the small stomach capacities of young children, common advice to parents is to provide regular meals and snacks 5–6 times throughout the day^(12,13)^, but little is known about the patterning of food intakes across the day among young children. Understanding the frequency of intakes, as well as their temporal distribution (i.e. how eating occasions are distributed or spread across a day by time), is valuable for identifying key opportunities across a day for nutrition promotion to improve dietary intake. Commonly, studies focus on total daily consumption, rather than considering eating frequency or distribution of food intake across the day as other important aspects of early childhood dietary behaviours. Studies of children’s intake distributions have shown evenings to have the highest energy intakes and contain the most vegetables^(14–18)^, whilst fruit and discretionary items were most commonly consumed in the afternoon^(16,17,19)^. However, these few existing studies examining child intake distributions have included a wide age range of children (8–16 year range); studies focused on those under 2 years are rare^(14–19)^, despite recognition that intake changes rapidly during this time^(20)^. Furthermore, existing studies have mostly examined overall eating frequency or energy intake distribution rather than the distribution of food groups. Understanding the temporal distribution of vegetable, fruit and discretionary food intakes may help identify potential opportunities for nutrition promotion messages.

While identifying opportunities to decrease intakes of discretionary foods and increase intakes of vegetables and fruits is of particular interest, it may also be important to consider these in the context of weekday *v*. weekend day intakes, and the patterning of energy intake and energy density. Two studies of older children (3–9 years) have found higher discretionary consumption and greater overall energy intake on weekends^(21,22)^ and higher consumption of fruits and vegetables on weekdays^(21)^. Given there may be different factors influencing intakes in earlier years, weekday–weekend differences in young children’s food intake patterns warrant investigation. Energy density considers foods and beverages consumed in combination and is likely associated with childhood obesity^(23,24)^. Discretionary foods and vegetables/fruits tend to be at opposing ends of the energy density spectrum, and so understanding the patterning of energy intake and energy density together with these food groups across the day may also be valuable^(25)^.

Eating more often should theoretically lead to higher overall intakes, for individual food groups and for total energy intakes, and consequently higher BMI could be expected^(18,26–28)^. Testing these assumptions is relevant to better understanding the complexities and mechanisms of child intakes and ultimately influences on BMI, which require further investigation for young children. Studies in older children largely suggest that a higher eating frequency is positively associated with BMI^(26)^, whilst a study of 2-year-old children found no such association^(29)^.

Therefore, the primary aim of the study was to describe the eating frequency and distribution of energy intake, energy density, vegetables, fruit and discretionary intakes across a day in 18-month-old children living in Australia. Additional aims were to assess differences between weekday and weekend days, and whether eating frequency was associated with intakes of relevant food groups and zBMI.

## Methods

### Study design and participants

This study utilised data from the 18-month-old data collection point in the Melbourne Infant Feeding Activity and Nutrition Trial (InFANT) Program: an obesity-prevention randomised controlled trial delivered to first-time parents from child age 3–18 months^(30)^. Participants were recruited in 2008 from first-time parents’ groups that were run within local government areas across Melbourne, Australia^(30)^. Programme inclusion criteria were that parents were English-speaking and first-time parents, participating in a selected first-time parents’ group. At baseline, 542 children and their families participated, with the sample size determined by sample size calculations for the Melbourne InFANT Program’s primary outcome: child vegetable intake^(30)^. Families in the control group received the usual care from routine Victorian Maternal and Child Health services, which includes general advice regarding infant feeding. Families in the intervention group additionally received six group sessions delivered by a dietitian over 15 months. At intervention conclusion, when the children were aged 18 months, 492 families remained^(30)^. No intervention effect at 18 months was observed for fruit or vegetable intakes, though there was a small difference in sweet snack intake^(30)^. Ethics approval was granted by Deakin University (EC 175- 2007) and the Victorian Office for Children (CDF/07/1138).

### Assessment of Covariates and BMI z-score

At the 18-month follow up, trained staff measured height and weight using standard protocols^(30)^, portable stadiometer (Invicta IPO955) and digital scales (Tanita 1582). Age- and sex-specific BMI z-scores were calculated using the WHO growth standards^(31)^. Child and maternal characteristics were assessed via self-reported survey completed at home at baseline (maternal education, maternal pre-pregnancy BMI, child sex (boys *v*. girls)) and 18-month follow up (child age (months))^(30)^. Maternal education was categorised into three groups: secondary school or below (usually ≤ 13 years of education), diploma or certificate qualifications (usually 13·5–15 years of education), and tertiary education (usually ≥ 16 years education)^(30)^. Maternal pre-pregnancy BMI was calculated as weight/height^2^ (kg/m^2^) ^(30)^. Treatment arm was also included as a covariate. These covariates were chosen as they are known to be associated with child intakes and/or zBMI for young children^(2,4,29)^.

### Assessment of dietary intake

Dietary intakes of children at age 18 months were assessed using three telephone-administered multiple-pass 24-h recalls with a parent^(2,32)^. Purpose-designed food measurement booklets assisted parents with quantity estimates. Dietary data were collected by purpose-trained researchers with nutrition qualifications and entered into a purpose-designed in-house database. Coding of all recalls was checked for accuracy and completeness by a dietitian, and quantities were imputed in the rare cases where quantity information was missing or deemed implausible by expert consensus (0·007 %). Recalls were conducted over two non-consecutive weekdays and one weekend day, including reporting of consumption time for each item. Dietary data were coded using the 2007 Australian Food and Nutrient Database (AUSNUT), and individual food and beverage items were matched with the respective nutrient composition to derive food, energy and nutrient intakes^(30)^. The present study analysed one weekday (the weekday closest to anthropometric data collection date) and one weekend day.

The intakes of vegetables (grams), fruits (grams) and discretionary foods (kilojoules) were the primary variables of interest in the present analyses, with the units of measurement based on how these foods are reported in the Australian Dietary Guidelines^(20)^. In the calculation of vegetable and fruit intakes, a disaggregation approach was used to ensure that proportions of mixed dishes that included vegetables and fruits were included^(2)^. The vegetable group consisted of all raw and cooked vegetables. For the fruit group, all raw, cooked, tinned and dried fruits were included, except juice^(2)^. Juice was classified as discretionary rather than as fruit or vegetables in line with emerging evidence^(33–35)^ and international guidelines^(9,36)^, that fruit juice has no essential role in healthy child diets, is associated with some health risks, and that it should only be used occasionally as whole fruit is preferable to juice. Items that were part of the discretionary food group were based on the classification established by Rangan and colleagues in addition to the examples listed by the Australian Guide to Healthy Eating^(2,20,37)^. Some examples include cakes, confectionery, pastries, processed meats, sugar-sweetened beverages and fruit juice. Energy (kilojoules) was used as the unit of measurement for discretionary foods in line with the serve definition in the Australian Dietary Guidelines^(20)^. Individual contributing items for each of these food groups were then summed.

Energy density was calculated based on two definitions. The first definition was ‘food-only’, which included all solid and liquid foods but excluded all beverages. The second definition was ‘food and caloric beverages’, which included all solid and liquid foods, dairy and dairy-substitute-based beverages, dairy beverage flavourings, infant/toddler formula and breast milk, and all other energetic beverages containing at least 21 kJ/100 g^(25,38,39)^.

### Eating occasions

Eating occasions were created based on the time of food intake reported, and combining any foods eaten < 15 min apart^(40–42)^. All energy-containing eating occasions were examined, and no minimum energy value was applied due to children’s young age and the possibility of eating occasions with small intakes^(43)^. Testing within the dataset showed that 1663 food items would have been excluded if the commonly used criteria of 210 kJ minimum intake for an eating occasion^(40)^ was applied. Eating frequency was calculated as the count of total eating occasions. This was repeated for eating occasions containing fruit, vegetable and discretionary food groups, to assess daily frequency of eating occasions containing foods from each food group. To calculate the sample average, non-consumers of the food group were included.

### Temporal distribution of energy intake, energy density and vegetable, fruit and discretionary food intakes

The mean total energy intake for each day and mean energy intake per hour was calculated using the same eating occasion definitions described above, including all eating occasions which started within each hour of the day. This analysis was conducted separately for weekdays and weekend days. Similarly, mean intakes of each of the assessed food groups (vegetables, fruits and discretionary foods) for each hour were calculated, as was the percentage of children consuming each food group within each hour. Energy density was also calculated for each hourly interval, for each of the two definitions provided, as the energy intake (kJ) in an hour divided by gram intake in that hour to obtain energy density (kJ/g) per hour.

### Statistical analysis

Due to rapid changes in diets that occur at this age, participants who were more than 6 months on either side of the mean age were excluded (*n* 1)^(30)^. Children who had daily energy intakes ±3 sd away from the mean energy intake were identified as outliers and excluded (*n* 6 for both weekdays and weekend days) ^(30)^.

All analyses were conducted in STATA IC 16^(44)^ with statistical significant difference set at *P* < 0·05 (two-sided). To analyse temporal distribution of intake, the hourly mean intakes of energy (kJ), energy density (kJ/g), fruit (g), vegetable (g) and discretionary foods (kJ) were calculated and graphed across a 24-h day, for each of weekdays and weekend days. When describing hourly intervals, the starting time of the hour was used (e.g. the interval 12.00–12:59 is written as 12.00). To compare weekday and weekend day intakes, multivariable linear regression was conducted across the same hours between weekdays and weekend days with adjustment for the covariates treatment arm, maternal education, maternal pre-pregnancy BMI, child sex, child age and child total weekday energy intake (kJ/d) (e.g. comparing 12.00–12:59 weekday mean with 12.00–12:59 weekend day mean, across each of 24 h). All participants who provided data for either weekday or weekend day were included in graphs and comparisons. This approach was verified by also repeating analyses with only complete cases (those with data for both weekday and weekend day, *n* 384), which showed no substantive differences from the original approach (data not presented).

Associations between eating frequency (total and each food group) and total intakes were assessed using multivariable linear regression analyses with the aforementioned maternal and child characteristics as covariates. To test the association between eating frequency (total and each food group) and zBMI at 18 months, multivariable linear regression was also conducted with the same covariates plus child total weekday energy intake. These analyses were undertaken for eating frequency on weekdays only given the minimal differences from weekend day intakes.

### Sensitivity analysis

As the InFANT Program was a randomised controlled trial, a sensitivity analysis was conducted for the descriptive analyses to test for differences in dietary intakes that may exist between the intervention and control groups.

## Results

**Fig. 1.**
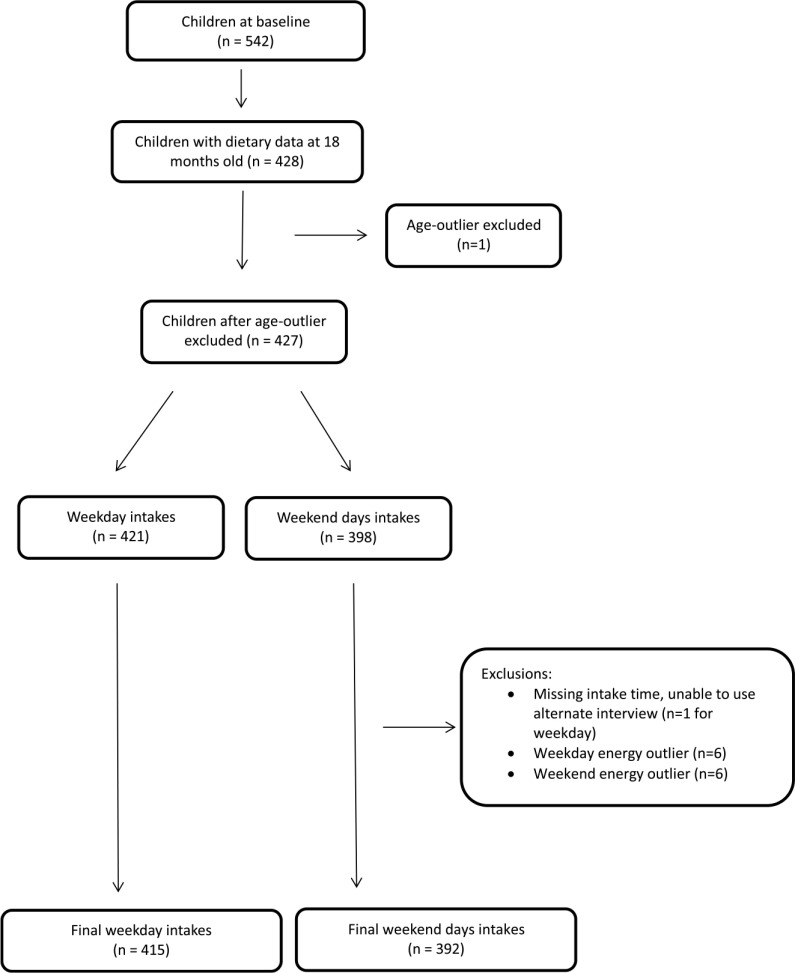
Flow chart outlining sample of the InFANT Program providing data for this analysis.

Of the 542 children recruited to the Melbourne InFANT Program at baseline, 427 children had dietary recall data at the 18-month-old follow-up (Fig. 1). After exclusions, 415 participants had included weekday data and 392 participants had weekend day data (Fig. 1). The mean age of children at the date of their first recall was 18·1 months ( ±1·5 months) and 55 % were male (Table 1). Over half of mothers had a tertiary education qualification.

**Table 1.** Demographic characteristics of participants in the Melbourne Infant Feeding Activity and Nutrition Trial (InFANT) Program, 2008–2010

Sample characteristic	*n*	%	Mean	sd
Age (months)	427		18·1	1·5
Sex (*n* 424)				
Male	231	55		
Female	193	45		
BMI z-score at 18 months	424		0·8	1·0
Treatment (*n* 424)				
Control	210	50		
Intervention	214	50		
Maternal education (*n* 424)				
Secondary school or below	87	20		
Diploma/certificate	100	24		
Tertiary education	237	56		
Maternal pre-pregnancy BMI (kg/m^2^)	424		24·9	7·9

Table 2 presents the average number of daily eating occasions and food group eating occasions on weekdays and weekend days. Out of the 7·8 eating occasions for weekdays, an average of 2·2 eating occasions included fruits, 1·5 occasions included vegetables and 2·5 occasions included discretionary foods (Table 2). No statistically significant differences were found between weekday and weekend day frequencies.

**Table 2.** Frequency of total and food group eating occasions on weekdays (*n* 415) and weekend days (*n* 392) of children aged 18 months in the Melbourne Infant Feeding Activity and Nutrition Trial (InFANT) Program, 2008–2010

Eating occasions	Weekday			Weekend day		
	Mean	sd	% consumers	Mean	sd	% consumers
Total	7·8	1·8	100·0	7·7	1·8	100·0
Vegetable	1·5	0·8	92·2	1·4	0·8	87·9
Fruit	2·2	1·1	95·2	2·2	1·2	91·6
Discretionary	2·5	1·5	94·1	2·6	1·4	95·0

No statistically significant differences were found between weekday and weekend day frequencies.

**Fig. 2.**
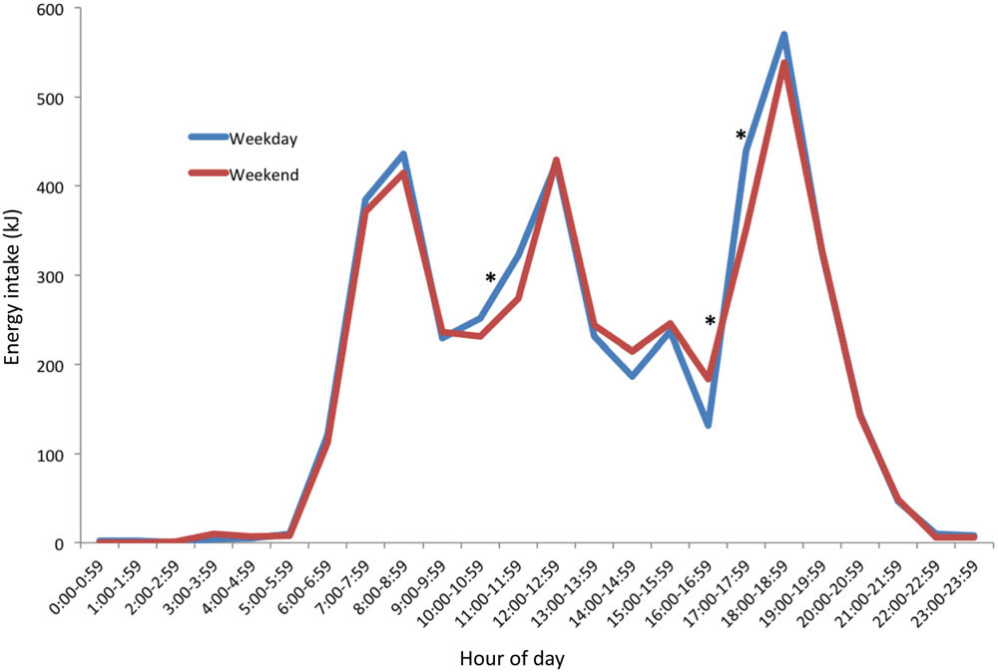
Distribution of hourly mean energy intake by weekday (*n* 415) and weekend days (*n* 392) of 18-month-old children. *Hour with statistically significant differences (*P* < 0·05) between weekday and weekend day.

Distribution of mean hourly energy intake on weekdays *v*. weekend days is presented in Fig. 2. Three distinct peaks occurred at 08.00, 12.00 and 18.00 for both weekdays and weekend days. On weekdays on average, 29 ± 12 % of energy intake occurred in a typical ‘dinner’ window (17.00–20.00), with ‘breakfast’ providing the next largest intake (21 ± 12 % from 06.00–09.00), followed by ‘lunch’ (17 ± 12 % from 11.00–13.00). In between the three peaks from 09.00 to 18.00, mean intake was above 150 kJ per hour across the day. Significant differences in energy intake between weekdays and weekend days were found at three times, with weekday intake higher at 11.00 (mean difference: 62·7, 95 % CI: 5·08, 120·3) and 17.00 (mean difference: 92·5, 95 % CI: 27·2, 157·9), and lower at 16.00 (mean difference: −48·9, 95 % CI: −89·0, −8·7).

**Fig. 3.**
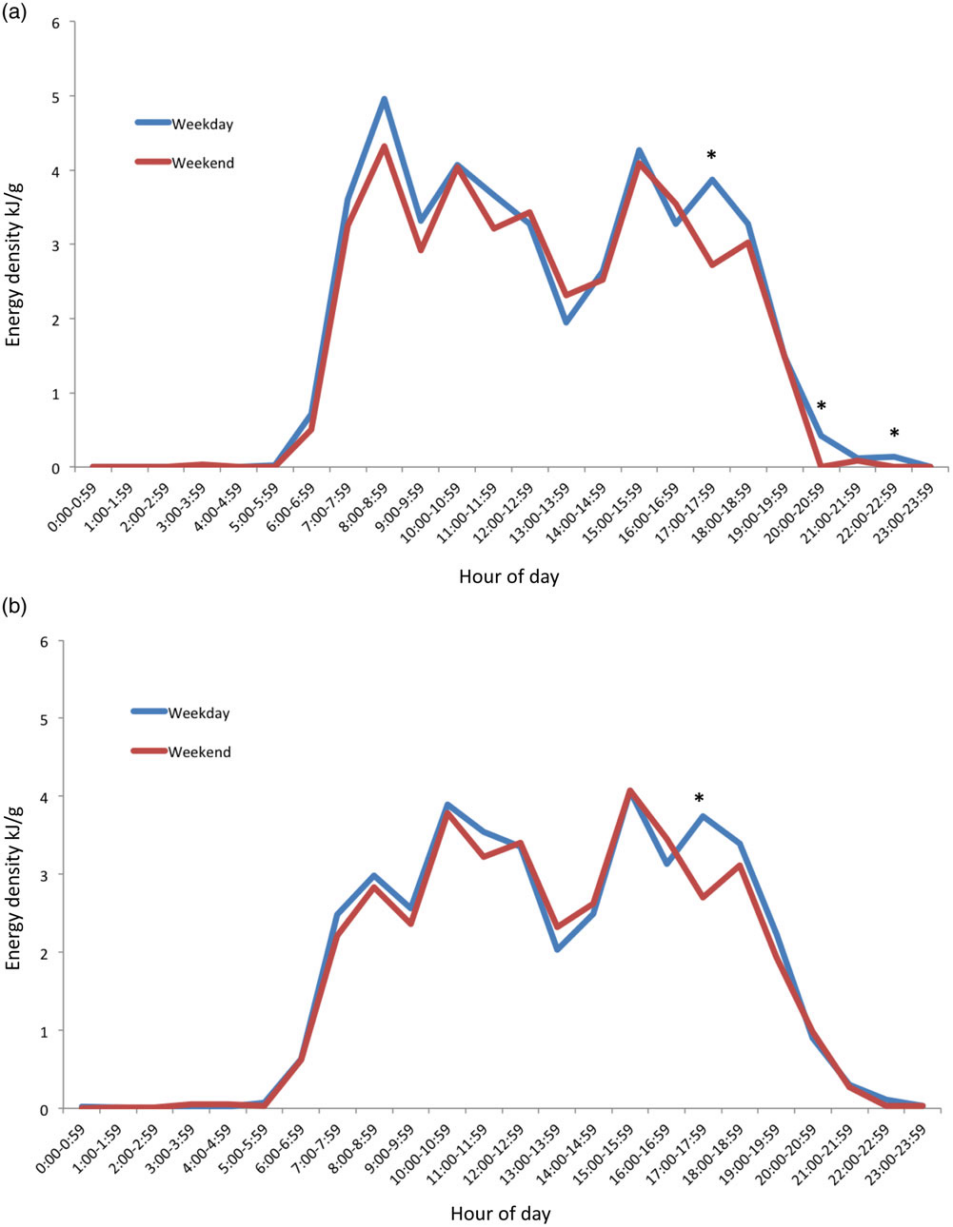
Distributions of hourly mean energy density across a 24-h period for weekday (*n* 415) and weekend days (*n* 392) of 18-month-old children. (a) Energy density of food-only, and (b) energy density including food and energy-containing beverages. *Hour with statistically significant differences (*P* < 0·05) between weekday and weekend day.

The consumption period for mean hourly food-only intakes (Fig. 3(a)) occurred between 05.00 and 23.00 on weekdays, and 05.00 to 21.00 on weekend days. Significantly higher weekday compared with weekend day energy density per hour was found at three times of the day. At 17.00, the mean difference was 1·1 kJ/g. (95 % CI: 0·5, 1·7). At each of 20.00 and 22.00, the mean difference was < 1·0 kJ/g. When beverages were included together with food intakes (Fig. 3(b)), the timing of intakes was similar with lower levels of energy density across the day. Energy density (food and beverage) on the weekdays was significantly higher (*P* < 0·05) at 17.00 (mean difference: 1·01, 95 % CI: 0·4, 1·6) than weekend days.

**Fig. 4.**
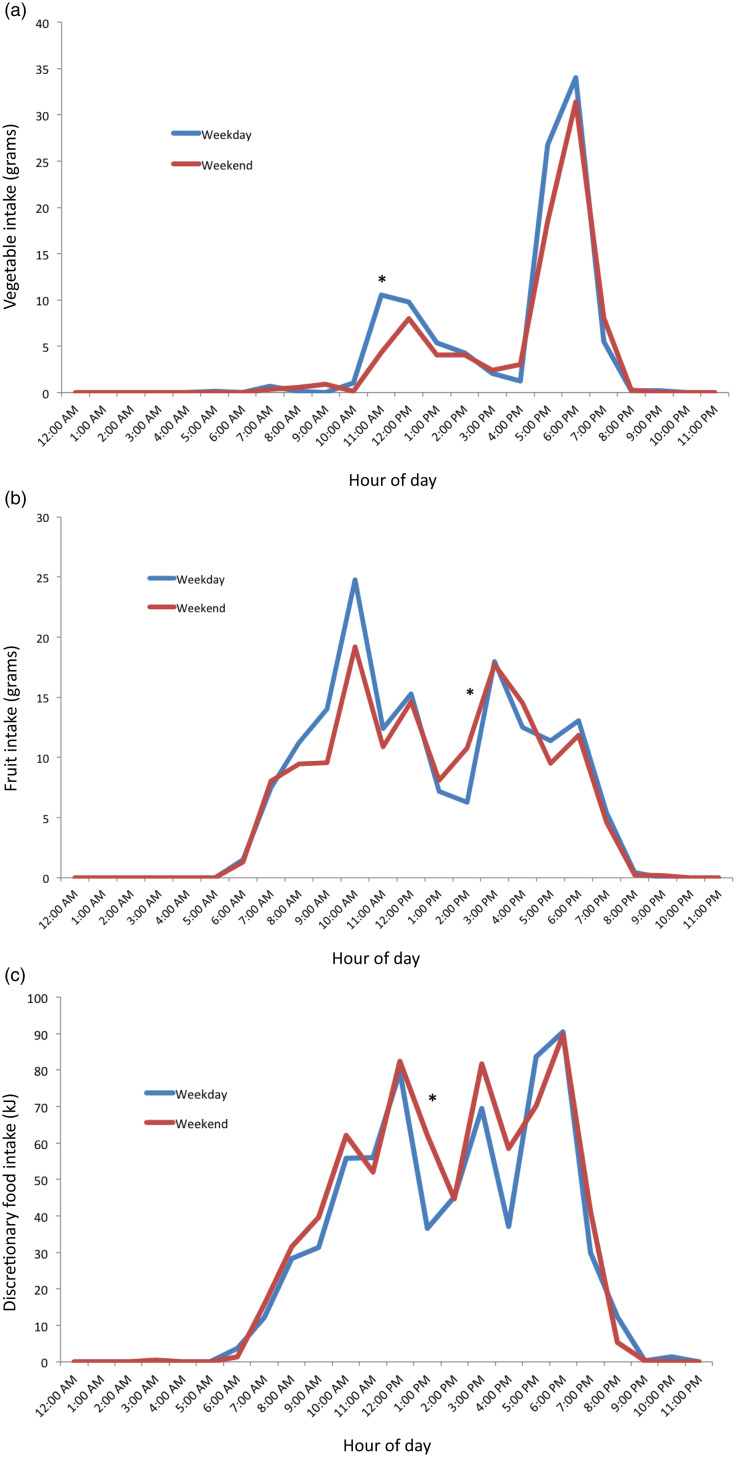
Distribution of mean hourly intake of food groups on weekday (*n* 415) and weekend (*n* 392) of 18-month-old children. (a) Vegetable, (b) fruit and (c) discretionary food groups. *Hour with statistically significant differences (*P* < 0·05) between weekday and weekend day.

Mean values of the distribution of intake for vegetables (g), fruit (g) and discretionary (kJ) food groups by weekday and weekend day are graphically represented in Fig. 4. Vegetable intake began at 07.00 for both weekdays and weekend days but was minimal except for two peaks at 11.00–13.00 and 17.00–19.00. The second peak at 17.00–19.00 represented substantially the largest intake across the day. This peak was 34 g on weekdays, which is about one-fifth of the daily recommendation of 150–225 g/d. A significant difference (*P* < 0·05) between weekday and weekend day intakes was found at 11.00 (mean difference: –6·6, 95 % CI: –11·0, –2·3) (Fig. 4(a)).

The consumption period for fruits began at 06.00 on both weekdays and weekend days. Four distinct peaks occurred from 10.00, 12.00, 15.00, and 18.00 for both weekdays and weekend days. These peaks reflected mean intakes of 13–25 g, representing one-fifth to one-third of the recommended daily intake of 75 g. Three distinct troughs also occurred in between these times. At 14.00, a statistically significant difference between weekday and weekend day fruit intakes (mean difference: 4·5, 95 % CI: 0·5, 8·8) was found (Fig. 4(b)).

Discretionary consumption began at 06.00, with three distinct peaks of similar magnitude at 12.00, 15.00 and 18.00 for both weekdays and weekend days. The mean intakes at these peaks were 70–90 kJ, in comparison with the recommendation of 0 kJ of such foods in this age group. Compared with weekend days, discretionary consumption on the weekdays was 33·4 kJ (95 % CI: 6·7, 60·1) lower at 13.00. (Fig. 4(c))

**Fig. 5.**
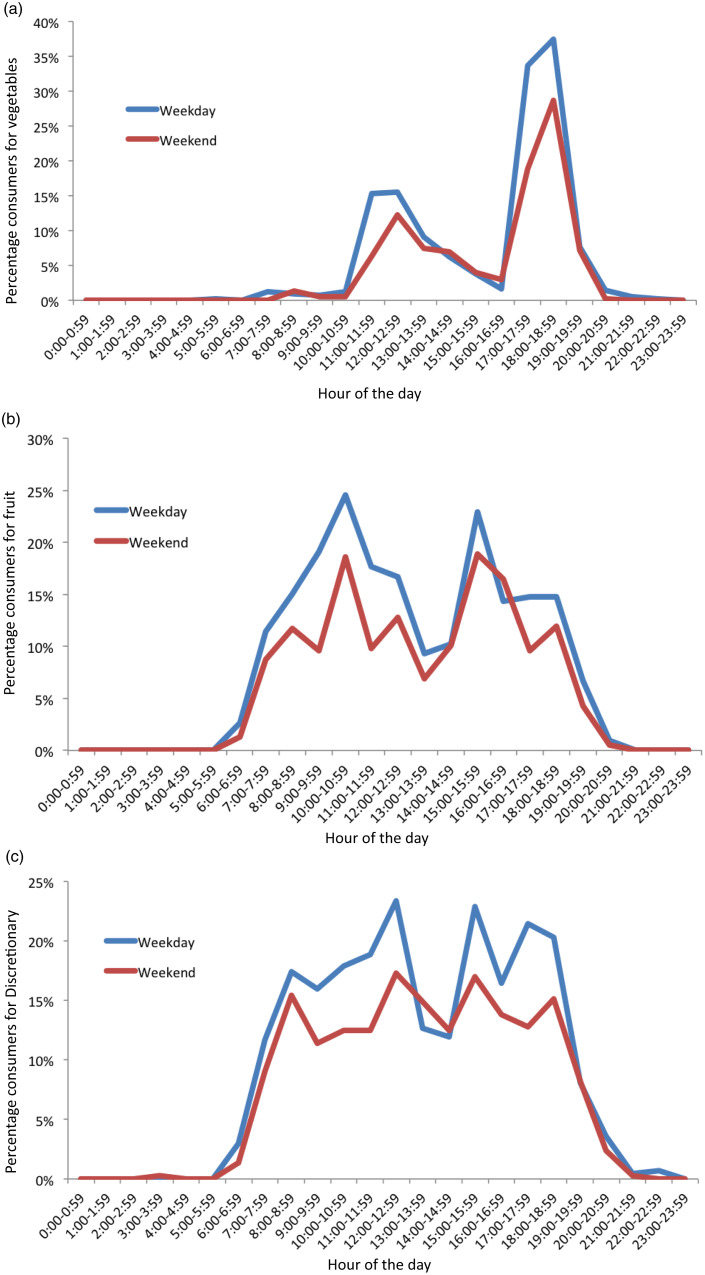
Percentage consumers for hourly intake of food groups on weekday (*n* 415) and weekend days (*n* 392) days of 18-month-old children. (a) Vegetable, (b) fruit and (c) discretionary food groups.

In Fig. 5, the percentage of consumers for hourly intake for each of the food groups on weekday and weekend days is graphically represented, showing similar results to Fig. 4.

### Association of eating frequency with total intakes and zBMI

Table 3 presents results on the association between frequency of weekday eating occasions of total foods, vegetables, fruits and discretionary foods, and total weekday daily intakes of those same food groups. The adjusted linear regression analyses identified positive associations (*P* < 0·01) in each instance. For example, every additional eating occasion was associated with an additional approximately 129 kJ of energy intake. For vegetables and fruits, every additional eating occasion was associated with an additional approximately 58 g of that food group intake.

**Table 3.** Association between total, fruit, vegetable, and discretionary EO and respective total intakes on weekdays for children aged 18 months in the Melbourne Infant Feeding Activity and Nutrition Trial (InFANT) Program, 2008–2010

Variables	Adjusted*		
	B-coef	95 % CI	*P*
Total EO (kJ)	129·4	69·03, 189·8	< 0·01
Vegetable EO (g)	57·12	46·20, 68·05	< 0·01
Fruit EO (g)	58·48	49·88, 67·08	< 0·01
Discretionary EO (kJ)	243·0	211·8, 274·2	< 0·01

EO, eating occasion.

* Multiple linear regression adjusted for treatment arm, maternal education, maternal pre-pregnancy BMI, child age and child sex.

No evidence of an association was found between frequency of weekday eating occasions and zBMI (Table 4). Similarly, frequency of fruit and vegetable eating occasions was not associated with zBMI. In contrast, frequency of discretionary eating occasions was inversely associated with zBMI (*P* = 0·04).

**Table 4.** Association between weekday EO and zBMI for children aged 18 months in the Melbourne Infant Feeding Activity and Nutrition Trial (InFANT) Program, 2008–2010

Variables	Adjusted*		
	B-coef	95 % CI	*P*
Total EO	–0·04	–0·09, 0·02	0·21
Vegetable EO	0·03	–0·12, 0·17	0·73
Fruit EO	–0·03	–0·14, 0·08	0·60
Discretionary EO	–0·09	–0·17, −0·00	0·04

EO, eating occasion.

* Multiple linear regression adjusted for treatment arm, maternal education, maternal pre-pregnancy BMI, child age, child sex and total weekday energy intake.

### Sensitivity analysis

The sensitivity analysis identified similar patterns of dietary intakes for control and intervention groups by weekday and weekend day (data not shown). No statistically significant differences were found between treatment groups for any time of the day on either weekdays or weekend days for fruit and vegetable intake. Discretionary intakes were significantly different for weekday intake at 13.00 only.

## Discussion

This study is the first to describe frequency and distribution of intakes for 18-month-old children for individual food groups. In particular, it provides a detailed understanding of the patterning of vegetable, fruit and discretionary food consumption. The most notable findings are the timing and low frequency of consumption of vegetables, demonstrated by only one main peak, and the consistent distribution of discretionary intakes throughout the day.

The frequency of daily eating occasions identified in the present study (7·8) is higher than what has been found in other research, where frequencies of 4·3–7 daily occasions have been reported in children aged between 1 and 11 years^(18,29,45)^. This is likely due to differences in age and methodology. For example, there is a lack of established definitions for characterising eating occasions in children, and previous studies have used varying and subjective definitions and wide time intervals spanning a few hours^(14,15,17,18,41)^. Such methods may not accurately capture young children’s eating patterns characterised by more frequent small eating occasions, such as identified in this study, due to rapid growth and smaller stomachs.

Vegetable intake was low overall, and the highest intakes were found in the evening, when ‘dinner’ (main meal) is typically consumed in Australia^(46)^. Our findings on vegetable eating occasions and timing of vegetable consumption are consistent with an Australian and a New Zealand study where dinner and evening periods (17.00–00.00), respectively, had the largest reported energy intakes and the highest percentage of consumers for vegetables^(16,19)^. Whilst a significant proportion of children in this study consumed vegetables between 17.00 and 19.00 (34–37 % per hour), the mean quantity in this time was small (e.g. highest graph peak between 18.00 and 19.00 showed mean intake of 34 g, or approximately one-fifth of the daily recommended amount of 150–225 g)^(20)^. This is particularly concerning given this was the only time of the day many children ate vegetables. Only one UK study in children aged 1·5–10 years has also assessed percentage of eating occasions including vegetables, and it reported similar findings to the present study^(17)^. The positive association between vegetable frequency and total vegetable intakes in this study is intuitive but important, as it shows that frequent consumption translates to overall greater intakes, not just the same daily amount distributed across more eating occasions. Taken together, these findings suggest an opportunity to increase quantity of vegetable consumption by either adding vegetables at additional eating occasions during the day (lunch, breakfast and snacks) or by increasing the amount consumed at the most popular eating occasion (dinner). This is in line with previous research finding many children consume vegetables only at dinner, suggesting a focus on other mealtimes namely lunch as a way to increase vegetable frequency and hence quantity^(19)^. Previously identified strategies such as role modelling, responsive feeding practices and eating together in meals could be used to support these approaches^(47,48)^.

Distribution of fruit and discretionary intakes in this study were characterised by intake across many hours in a day in addition to several peaks. Peak times for fruit and discretionary intakes were similar, which may indicate that these were commonly eaten as snacks consumed at similar time points, though not necessarily by the same children. A number of studies have identified that fruits, followed by discretionary foods, are commonly eaten throughout the day as snacks^(15,16,49)^. Though it was notable that the highest discretionary peak was in the dinner period, showing these foods are not just consumed as snacks and are also displacing more nutritious evening meal options. This frequent consumption of discretionary foods throughout the day is consistent with, and may explain, the high overall discretionary intakes reported in young children^(49,50)^. The present study findings suggest that interventions to reduce discretionary food frequency and quantity are warranted across the day, particularly given that frequency of intake was also found to be strongly associated with total intake quantities. Ideally, discretionary intakes could be displaced by vegetable or fruit intakes, to achieve mutually beneficial outcomes across food groups, though this poses practical challenges.

Conversely for fruits, the similar consumption pattern across the day, together with average frequency more than twice per day, and the association of frequency with total intake (previously reported to be adequate at this age)^(2)^, are positive findings for this food group. This suggests that fruit may need less priority in nutrition promotion initiatives for this age group and reinforces the need for different nutrition promotion messaging across individual food groups. Fruit and vegetable intakes are typically studied and promoted together, but it is clear from this study and other research that vegetable intakes require more focus for promotion^(51)^.

Research assessing differences in dietary intakes between weekdays and weekend days in young children is rare. In contrast to three studies in older children aged 2–9 years that found weekends contributed to greater amounts of energy intakes and frequency of eating occasions than weekdays^(21,52,53)^, the present study revealed no difference in energy intake and eating frequency between weekdays and weekend days among 18-month-old children. This could be due to daily schedules during toddlerhood being more similar across weekdays and weekend days and largely determined by their primary caregiver^(54)^. It may be that weekend day compared with weekday is less distinguishing than other changes to routine in this age group, such as childcare *v*. non-childcare days. Few children in this study attended full-time childcare or recorded recalls about days in childcare.

Examination of energy density using two approaches from the literature^(23,55)^ showed similar patterns of consumption between 07.00 and 19.00 with consistent mean levels throughout the day. Patterns of energy density most closely resemble those for distribution of discretionary intakes, suggesting that this food group may be a key factor in determining energy density spread across a day. This is in line with other studies’ findings of the substantial contribution and frequency of discretionary foods in diets and energy intakes of children aged 1–18 years^(2,49,50,56,57)^. Energy intake was highest in the dinner period, also in line with other studies of Australian and New Zealand children^(14,16)^.

When investigating associations of eating frequency with zBMI, the majority of studies focus on total eating frequency rather than examining individual food groups^(26)^. This present study found a small inverse cross-sectional association between discretionary eating occasions and zBMI. This unexpected finding may be due to parents of children with obesity being likely to report in a socially desirable way, or young children self-regulating energy intake or exhibiting compensatory behaviour (as physical activity and energy expenditure were not assessed in this study)^(58)^. It is also possible that child zBMI has bidirectional influence on intake; for example, carers of children with lower zBMI may be less controlling of their child’s discretionary food intakes^(26)^. Previous studies investigating eating frequency and zBMI found no associations in young children aged 2–3·5 years^(29)^, whilst in older children, results are mixed^(18,26,27)^. A robust explanation for this relationship has not been provided in current literature^(29)^; however, these findings seem to indicate that associations between eating frequency and zBMI present more as age progresses. Regardless of early-life cross-sectional associations between intakes and zBMI, intakes at this age are likely to impact on the establishment of food preferences and may be more important in influencing later dietary intakes and health outcomes.

Strengths of this study include investigation of an under-studied age group, namely children under 2 years of age. The data are from multiple 24-h recalls based on a methodology validated by the US Department of Agriculture and a disaggregation approach with dietary data to most thoroughly capture fruit and vegetable intakes that is not ubiquitous across many studies^(2,59)^. The dataset also covers both weekdays and weekends for most participants. Additionally, this study provides descriptive data on specific hourly intakes for fruits, vegetables, discretionary foods, energy density and energy intake. Clear definition for determining eating occasions, and conversion into hourly time intervals, was also a strength as this allowed for a data-driven approach rather than the use of subjective mealtimes, wider time intervals (e.g. 3 h, during which time a young child might consume two meals), or researcher-defined timings, which have been used in some existing literature^(16,18)^.

It was beyond the scope of this study to assess all food groups, but future research with inclusion of the other food groups could facilitate understanding of total meal patterns including how different food groups are consumed together. For example, a New Zealand study identified that vegetables were consumed often with meat and potato/taro foods and that study proposed a strategy to increase vegetable consumption by including it in combination with meat and potato^(16)^. As well as other food groups, in future research, it would also be interesting to analyse eating frequency among this age group against meeting nutrient requirements.

Limitations include those inherent to 24-h recall data, namely, not necessarily representative of usual intake, not taking into account foods that are consumed episodically^(60)^, recall bias and social desirability bias^(61)^. Also, the method used to collect dietary data did not assess food provision, only consumption. While young children are learning to eat and try new foods, and where responsive feeding principles are followed, what foods are offered and role modelled may be as important as what foods are consumed^(48)^. Assessing food provision and concurrent consumption of adults present, as well as child consumption, could be a direction for future research on mealtime opportunities for nutrition promotion. Another limitation is the use of a dataset obtained from a randomised controlled trial. However, given the aims of this analysis were assessing distribution and frequency rather than total intakes, inclusion of participants from both intervention and control arms was considered appropriate for a robust sample size^(2)^. To address this, sensitivity analyses were conducted with minimal differences identified. Finally, participants had a relatively high maternal education and findings may therefore not be generalisable to the broader Australian population, given the established associations between child diets and maternal education^(54)^. Taken together, these sample characteristics may suggest findings to be reflective of a best-case scenario; however, future research in larger and more representative samples is warranted.

### Conclusion

Findings of food group distribution and frequency in 18-month-old Australian children complement existing evidence on inadequacies in total dietary intakes by showing timing of intakes more specifically. In particular, vegetable consumption mostly occurs at the evening meal but has low frequency across the rest of the day, in contrast to intakes of fruit, discretionary foods and energy, which are distributed across the day. Interpretations to inform further research and practice include that vegetable intakes could be improved with promotion of intakes outside of evening times, and discretionary intakes could be reduced throughout the day. Targeting the way in which children and families eat in meals and snacks may provide more salient messages that provide practical strategies for nutrition promotion.
